# Norepinephrine promotes tumor microenvironment reactivity through β3-adrenoreceptors during melanoma progression

**DOI:** 10.18632/oncotarget.2652

**Published:** 2014-12-06

**Authors:** Maura Calvani, Floriane Pelon, Giuseppina Comito, Maria Letizia Taddei, Silvia Moretti, Stefania Innocenti, Romina Nassini, Gianni Gerlini, Lorenzo Borgognoni, Franco Bambi, Elisa Giannoni, Luca Filippi, Paola Chiarugi

**Affiliations:** ^1^ Department of Experimental and Clinical Biomedical Sciences, University of Florence, Tuscany Tumor Institute and “Center for Research, Transfer and High Education DenoTHE”, Florence 50134, Italy; ^2^ Plastic Surgery Unit, Regional Melanoma Referral Center, Tuscan Tumor Institute, Santa Maria Annunziata Hospital, Florence 50012, Italy; ^3^ Department of Surgery and Translational Medicine, Dermatology Section University of Florence, Florence, Italy; ^4^ Division of Pathology, Pistoia Hospital, Pistoia, Italy; ^5^ Department of Health Sciences, Clinical Pharmacology and Oncology Unit, University of Florence, Florence, Italy; ^6^ Neonatal Intensive Care Unit, Medical Surgical Fetal-Neonatal Department, “A. Meyer” University Children's Hospital, Florence, Italy; ^7^ Transfusion Medicine and Cell Therapy “A. Meyer” University Children's Hospital, Florence, Italy

**Keywords:** β-adrenergic receptors, melanoma, tumor microenvironment, cancer associated fibroblasts, macrophages, mesenchymal stem cells

## Abstract

Stress has an emerging role in cancer and targeting stress-related β-adrenergic receptors (AR) has been proposed as a potential therapeutic approach in melanoma. Here we report that β3-AR expression correlates with melanoma aggressiveness. In addition, we highlight that β3-AR expression is not only restricted to cancer cells, but it is also expressed *in vivo* in stromal, inflammatory and vascular cells of the melanoma microenvironment. Particularly, we demonstrated that β3-AR can (i) instruct melanoma cells to respond to environmental stimuli, (ii) enhance melanoma cells response to stromal fibroblasts and macrophages, (iii) increase melanoma cell motility and (iv) induce stem-like traits. Noteworthy, β3-AR activation in melanoma accessory cells drives stromal reactivity by inducing pro-inflammatory cytokines secretion and *de novo* angiogenesis, sustaining tumor growth and melanoma aggressiveness. β3-ARs also play a mandatory role in the recruitment to tumor sites of circulating stromal cells precursors, in the differentiation of these cells towards different lineages, further favoring tumor inflammation, angiogenesis and ultimately melanoma malignancy. Our findings validate selective β3-AR antagonists as potential promising anti-metastatic agents. These could be used to complement current therapeutic approaches for melanoma patients (e.g. propranolol) by targeting non-neoplastic stromal cells, hence reducing therapy resistance of melanoma.

## INTRODUCTION

Several studies suggest that behavioral-related factors as catecholamines, hormones released in response to stress from the sympathetic nervous system, accelerate cancer progression and decrease cancer patient survival [1]. Various biological effects of catecholamines in cancer cells have been associated to β-adrenergic receptors subfamily (β-ARs), composed of three members that signal through distinct downstream pathways [2, 3]. The three subtypes of β-ARs, β_1_, β_2_, and β_3_, are widely expressed in different tumors, such as those of the brain, lung, liver, kidney, adrenal gland, breast, ovary, prostate or lymphoid tissues [4–7]. We have recently reported that primary melanoma cells express both β1 and β2-ARs and that β2-ARs are up-regulated in metastatic melanoma, with a strong correlation with malignancy [4]. β3-AR plays a key role in the pathophysiology of the cardiovascular system and has been recently correlated with *de novo* angiogenesis in models of retinal vascular proliferation [8–10]. Moreover, β3-AR mRNA aberrant expression has been reported in human cancers, such as leukemia, vascular tumors and colon carcinoma [5, 11, 12]. Recently, β_3_-ARs has been found to be expressed by murine melanoma B16F10 cells and by endothelial cells of the tumor vasculature [13, 14]. Finally, beside deregulation of β_3_-ARs expression, Trp64Arg β_3_-AR polymorphism has been associated with susceptibility to endometrial and breast cancers [15, 16].

Although epidemiological data are still contradictory, preclinical studies suggest that β-blockers favorably impact on disease progression in several types of cancers, mainly by reducing metastases, tumor recurrence and mortality, [17–19]. In keeping with this, we have previously demonstrated a significant activation of pro-tumorigenic biological responses induced by catecholamines in melanoma cells, severely inhibited by propranolol, a non-selective inhibitor that can block β-ARs [4]. Although propranolol is able to impair key features of melanoma malignancy, such as proliferation, motility, secretion of metalloproteases, invasiveness and secretion of pro-angiogenic and pro-inflammatory cytokines, the specific contribution of β-ARs in controlling these cellular events is still unknown. The ability of catecholamines to induce in melanoma cells the expression of the pro-inflammatory and pro-angiogenic interleukin-6 (IL-6), interleukin 8 (IL-8) and vascular endothelial growth factor (VEGF) prompted us to study the role of β-AR functions within tumor microenvironment [20, 21]. Indeed, tumor progression is a multistep process controlled by the cross-talk between tumor and stromal cells. Stromal cells can be either resident or recruited to tumor site from circulating bone marrow precursors to sustain tumor growth and to orchestrate vasculogenesis, lymphoangiogenesis and inflammation [22–24]. The microenvironment supporting tumors progression is composed by endothelial cells, cancer associated fibroblasts and macrophages (CAFs and CAMs), tumor associated lymphocytes and neuthrophils [25–27]. Catecholamines are released locally by sympathetic nerve fibers or can be found circulating in the blood. Although β-ARs could be activated on both tumor and stromal cells by catecholamines, data on the role of these receptors within the tumor microenvironment are needed to develop innovative therapeutic approaches.

Herein we investigated the role of several cell populations that compose the melanoma microenvironment (i.e. melanoma-associated fibroblasts, macrophages, endothelial cells and bone marrow derived mesenchymal cells) during cancer progression. Our findings indicate a differential involvement of β2 and β3-ARs in the recruitment and differentiation of circulating precursors of stromal cells by the tumor. This recruitment sustains tumor inflammation, angiogenesis and ultimately promotes melanoma malignancy. Finally, our data validate selective β-blockers as effective drugs to target both autonomous and non-autonomous oncogenic pathways in advanced melanoma.

## RESULTS

### β3-ARs expression in tissue samples

We have previously described the role of β2-ARs in melanoma [4]. Here we wanted to address the role of β3-ARs expression in melanoma malignancy. Consequently, we investigated β3-ARs expression in a cohort of human samples of common melanocytic nevi (CN), atypical melanocytic nevi (AN), *in situ* primary melanoma (ISM), superficial spreading melanoma (SSM), nodular melanoma (NM) and cutaneous and lymph-nodal metastatic melanoma (MM). β3-AR was expressed, although at various levels, in all examined melanocytic lesions. The immunostaining of each group, taking into account both staining intensity and percentage of positive cells (both composing the score), is shown in Table 1. Score 1 was observed in all CN and AN but one (AN) which expressed score 2, and in all ISM but one, which showed score 2. Score 2 was detected in all SSM and NM but one NM, which exhibited score 3. MM showed score 3 in four cases and score 2 in six cases. β3-AR score was significantly higher in malignant lesions compared to nevi (*p* = 0.000068). ISM + SSM exhibited a significantly lower score compared to NM+MM (*p* = 0.0087), and no difference was observed between CN and AN. The cell staining intensity of melanocytic/melanoma cells was constantly weak, and moderate in only 3 metastases.

**Table 1:**
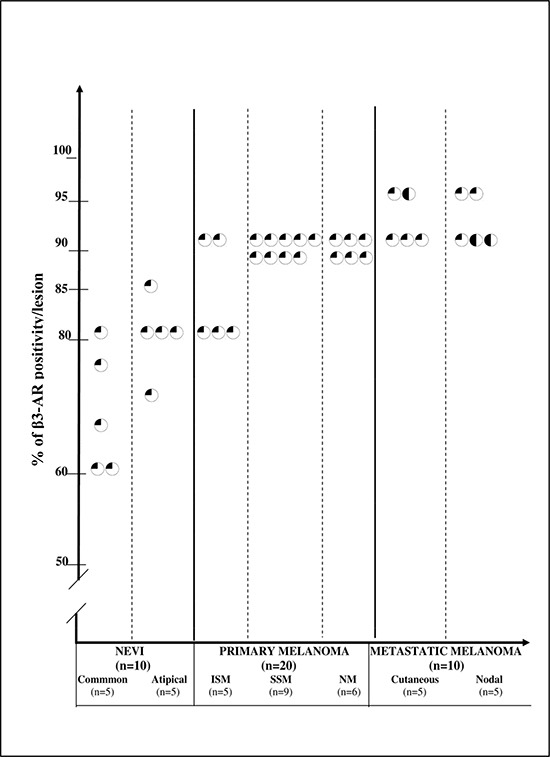
Immuno-histochemical expression of β3-AR in cutaneous human melanocytic lesions: percentage of positivity in each lesion Each circle represents the percentage of stained cells for one lesion. A quarter-black circle indicates positive weak staining; a half-black circle indicates positive moderate staining; a three-quarter-black circle indicates positive strong staining; a solid-black circle indicates very strong staining.

**Figure 1:**
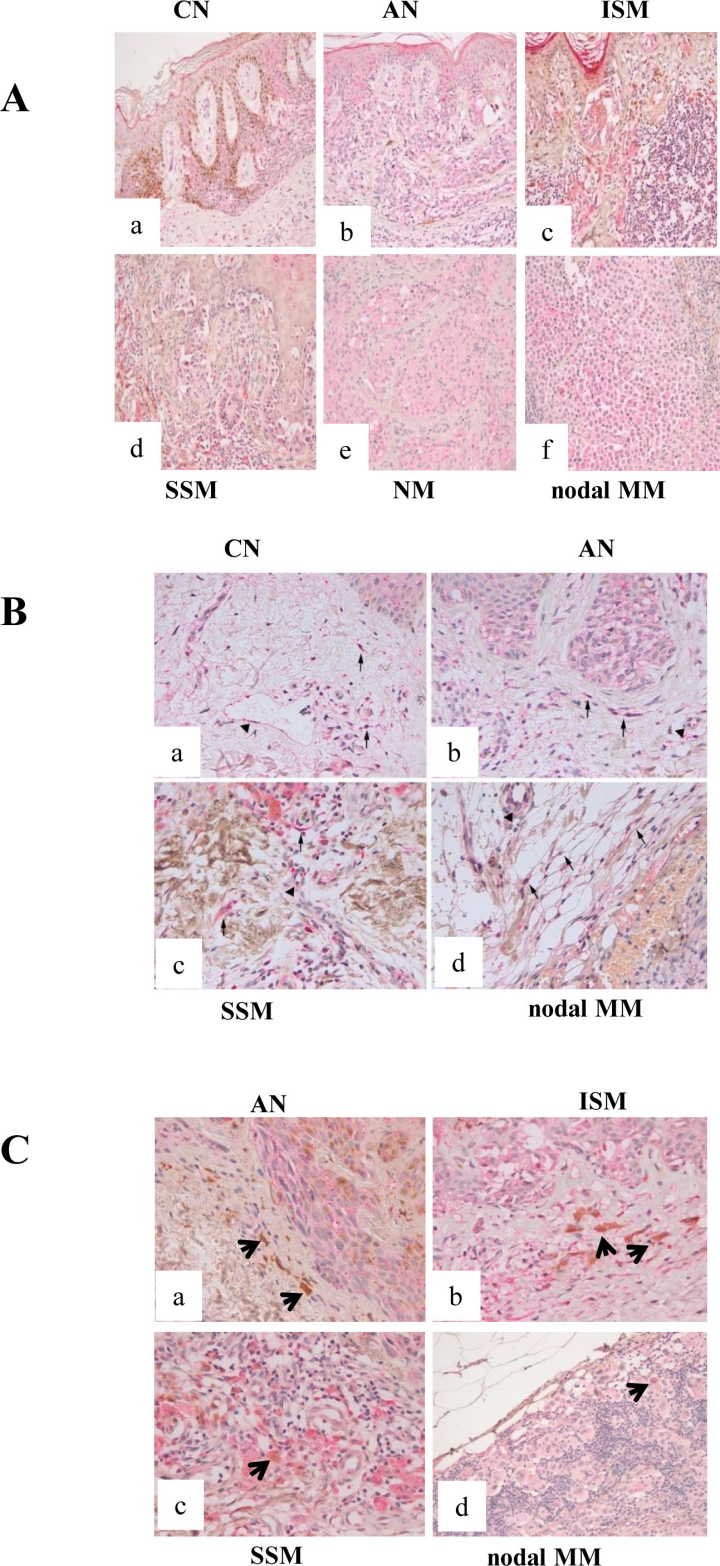
β3-ARs expression in human samples **(A)** β3-ARs expression in human cutaneous melanocytic lesions (X200). β3-AR immunostaining in junctional CN (a), AN (b), ISM (c), SSM (d), NM (e), nodal MM (f): all lesions show a low reaction intensity, confined to the cell cytoplasm. Epidermal keratinocytes exhibit staining, with strong positivity in granular layer; stromal cells also show reactivity. **(B)** β3-ARs expression in the microenvironment of human cutaneous melanocytic lesions (X400). (a) normal skin adjacent to CN; (b) AN; (c) SSM; (d) nodal MM. Arrows point to positive fibroblasts and arrow-heads point to positive blood vessels. Epidermis and in particular granular layer show positive reaction. Benign and malignant melanocytes are also stained. **(C)** Expression of β3-ARs in the microenvironment of human cutaneous melanocytic lesions. (a) AN and (b) ISM show melanophages (arrows) negative for β3-AR. (c) SSM and (d) nodal MM exhibit macrophages positive for β3-AR staining. Benign and malignant melanocytes are stained.

Representative β3-AR IHC pictures of melanocytic lesions are shown in Fig. 1A. Beside melanoma cells, stromal cells show strong immunoreactivity. Granular layer of the epidermis, epidermal keratinocytes and dermal fibroblasts always showed strong positive reaction. Moreover, endothelial cells appeared to be stained in all sections. Macrophages and lymphocytic infiltrate exhibited reactivity only in invasive malignant lesions (Fig. 1B, C).

Taken together, our data show that β3-AR is expressed in human melanocytic lesions with a significant up-regulation in malignant and advanced malignant lesions. Importantly, β3-AR was expressed in stromal, endothelial and inflammatory cells (Fig. 1B, C).

### *In vitro* β3-AR expression is affected by hypoxia, nutrients and stromal cells contact

**Figure 2:**
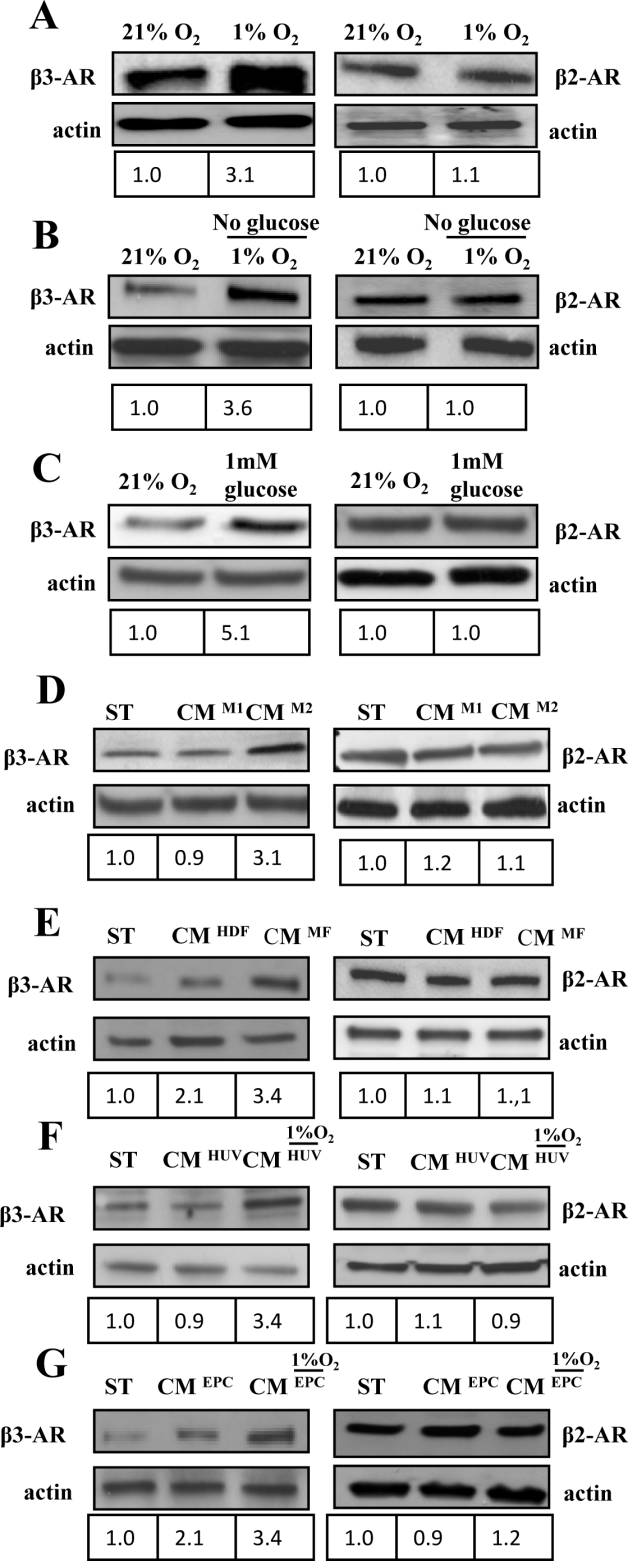
β-ARs expression in melanoma cells under various microenvironmental conditions **(A-G)** 1 × 10^6^ A375 human melanoma cells were serum starved for 24 h and subsequently treated as reported in figure for 24 h. β3 and β2 ARs expression was evaluated in total lysates by immunoblotting analysis. Cells were exposed to **(A)** hypoxia (1%O_2_); **(B)** ischemia (1%O_2_ and 0% glucose); **(C)** hypoglycemia (1 mM) or exposed to CM derived from different activated stromal cells of tumor microenvironment: **(D)** M1 or M2 macrophages; **(E)** HDFs stimulated with TGFβ (myofibroblasts, MFs), as previously reported [55] or **(F, G)**. HUVECs and EPCs exposed to hypoxic conditions. Anti-actin immunoblot was used to ensure equal protein loading. The figure is representative of three independent experiments.

Solid tumors initially develop in the absence of vascularization and are subjected to various growth constraints due to hypoxia and ischemia. This condition induces a pleiotropic cellular response that includes metabolic adaptation to change in nutrients and oxygen supply. We evaluated if β2/β3ARs expression in A375 human melanoma cells were affected from such conditions (i.e. hypoxia and ischemia). 1% oxygen concentration is used to mimic *in vitro* intratumoral hypoxia, changes in glucose supply to analyze nutrient sensing and the combination of the these factors to mimic ischemic conditions. Our results indicate that hypoxia, ischemia and glucose withdrawal lead to an upregulation of β3-ARs expression, while β2-ARs expression is almost unchanged (Fig. 2A, B, C).

Tumor microenvironment is also constituted by several accessory cells that may help and sustain melanoma progression. We studied the effect of several accessory cells, commonly present in solid tumors, on β2/3-ARs expression: i) macrophages polarized *in vitro* toward the antitumoral phenotype M1 or toward the pro-tumoral M2 phenotype [28]; ii) HDFs, activated *in vitro* by transforming growth factor-β1 (TGF-β) to behave as MFs; iii) HUVECs and EPCs, as endothelial precursors. Endothelial cells were also exposed to 1% O2 hypoxia and their conditioned medium was then used to treat A375 melanoma cells for 24 h to mimic intratumoral hypoxia. Our results reveal that accessory cells are not able to affect β2-ARs, but they are extremely successful in upregulating β3-ARs expression (Fig. 2D, E, F, G). In particular, HDFs, M2 macrophages and EPCs cells are the most efficient in enhancing β3-ARs expression levels, while exposure to hypoxia is able to strengthen the response of all accessory cells, including HUVECs (Fig. 2F).

The vast majority of cutaneous melanomas show activating mutations in BRAF proto-oncogenes, sustaining proliferative stimuli [29, 30]. A375 cell lines are therefore a representative model of such melanoma subtype, since they show BRAF V600E mutation (REF). In order to study the role of β-AR expression and activation in these cells, we examined the activation of the extracellular regulated kinase (ERK) pathway in response to NE stimulation, in the presence or absence of the β3-AR-blocker SR59230A, the β2-AR-blocker IC118551, as well as the non-selective β-AR-blocker propranolol. The results suggest that ligand stimulation of β-ARs by NE is able to enhance basal ERK1/2 activation. Importantly, all β-AR blockers tested revert this effect, although inhibition of β3-AR appears to be the most effective. In addition, we also confirmed these results by RNA interference of β2 and β3-ARs (Supplementary Fig. 1).

### β-ARs *in vitro* activation drives the recruitment of stromal cells to primary melanoma

**Figure 3:**
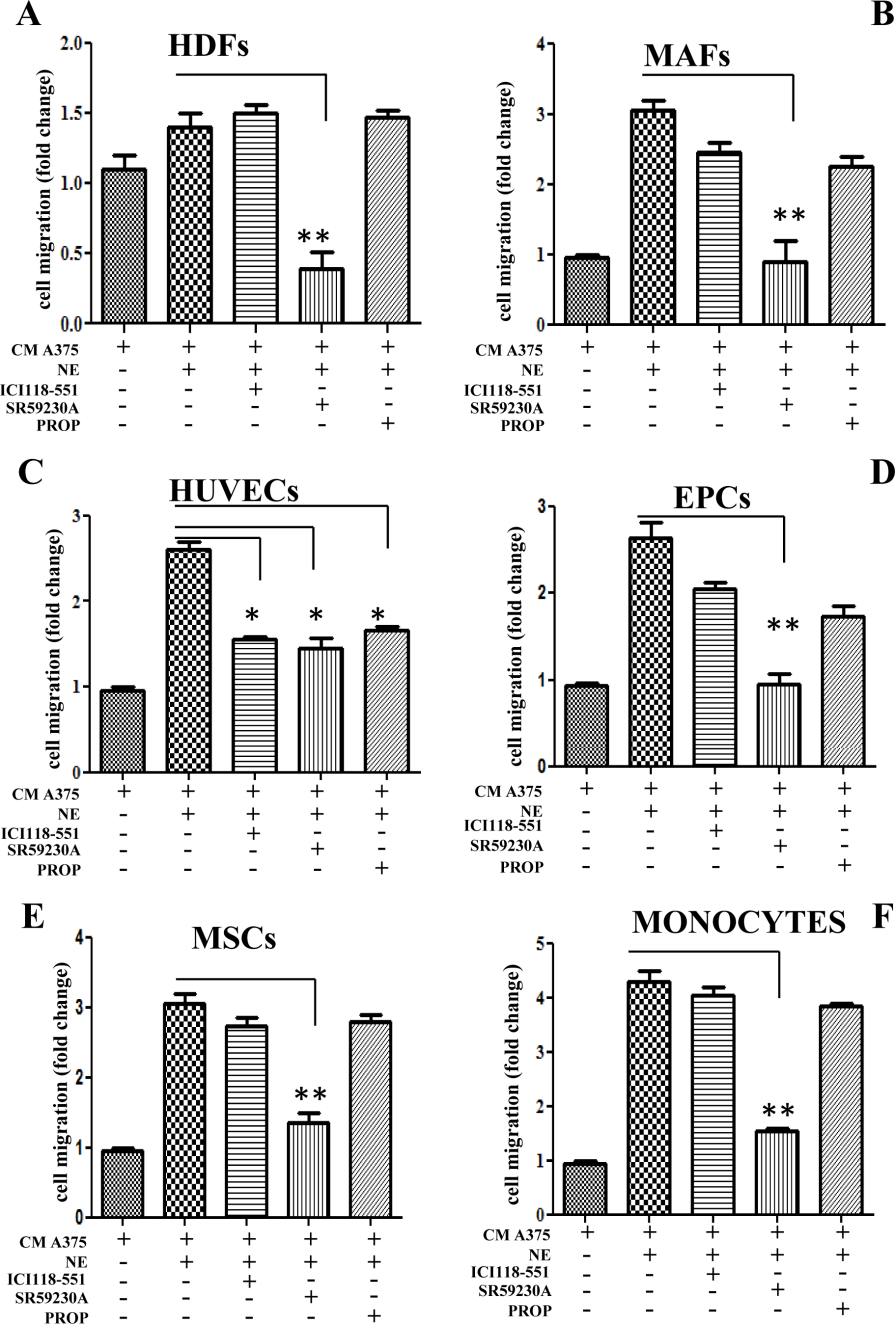
Recruitment of stromal cells Different stromal cells were allowed to migrate for 24 h toward CM derived from A375 melanoma cells. In left panels, A375 cells were incubated in the presence or absence of NE (10 μM) and/or β-ARs antagonist ICI 118-551 (1 μM), SR59230A (10 μM) and propranolol (1 μM). Figure shows recruitment of HDFs **(A)**; MAFs **(B)**; HUVECs **(C)**; EPCs **(D)**; MSCs **(E)** and monocytes **(F)**. The figure is representative of three independent experiments. *P < 0.05, **P < 0.001, ***P < 0.0001 *vs* NE stimulated cells.

The ability of primary melanoma cells to recruit accessory cells in response to ligand activation of β3-AR has been assessed by transwell chemotaxis assay. We used HDF and fibroblasts derived from human primary melanoma (MAFs). Conditioned medium derived from A375 cells stimulated with norepinephrine (NE) for 24 h elicits an increase in recruitment of HDFs, MAFs, HUVECs, as well as MSCs, EPCs and monocytes (Fig. 3) when compared to conditioned medium derived from unstimulated A375. Ligand stimulation is a mandatory step for these recruitments. Indeed, to definitely identify the differential role of β2 and β3-ARs, we used two different approaches; a pharmacological one by treating cells with selective β-AR blockers (i.e. ICI118-551 for β2-AR and SR-59230A for β3-AR) or by RNA interfering with the two β-ARs (Supplementary Fig. 2). The results suggested a role of β3-AR in recruiting not only fibroblasts (both HDFs and MAFs) but also MSCs and monocytes (Fig. 3A, B, E, F). Conversely, β3-AR and β2-AR have similar the recruiting abilities towards endothelial cells (Fig. 3C, D). Accordingly, only high doses of propranolol are active on recruitment of stromal cells (Supplementary Fig. 3). To exclude a possible role of α1-ARs in attracting stromal cells *in vitro*, we performed HDFs and HUVECs recruitment by conditioned medium of NE-treated A375 cells, using the selective α1-adrenergic antagonist prazosin. As expected, prazosin has no effect on both HDFs and HUVECs recruitment by NE-treated melanoma cells (Supplementary Fig. 4A-B).

### β3-AR drives accessory cells reactivity and enhances melanoma:fibroblasts cross-talk

**Figure 4:**
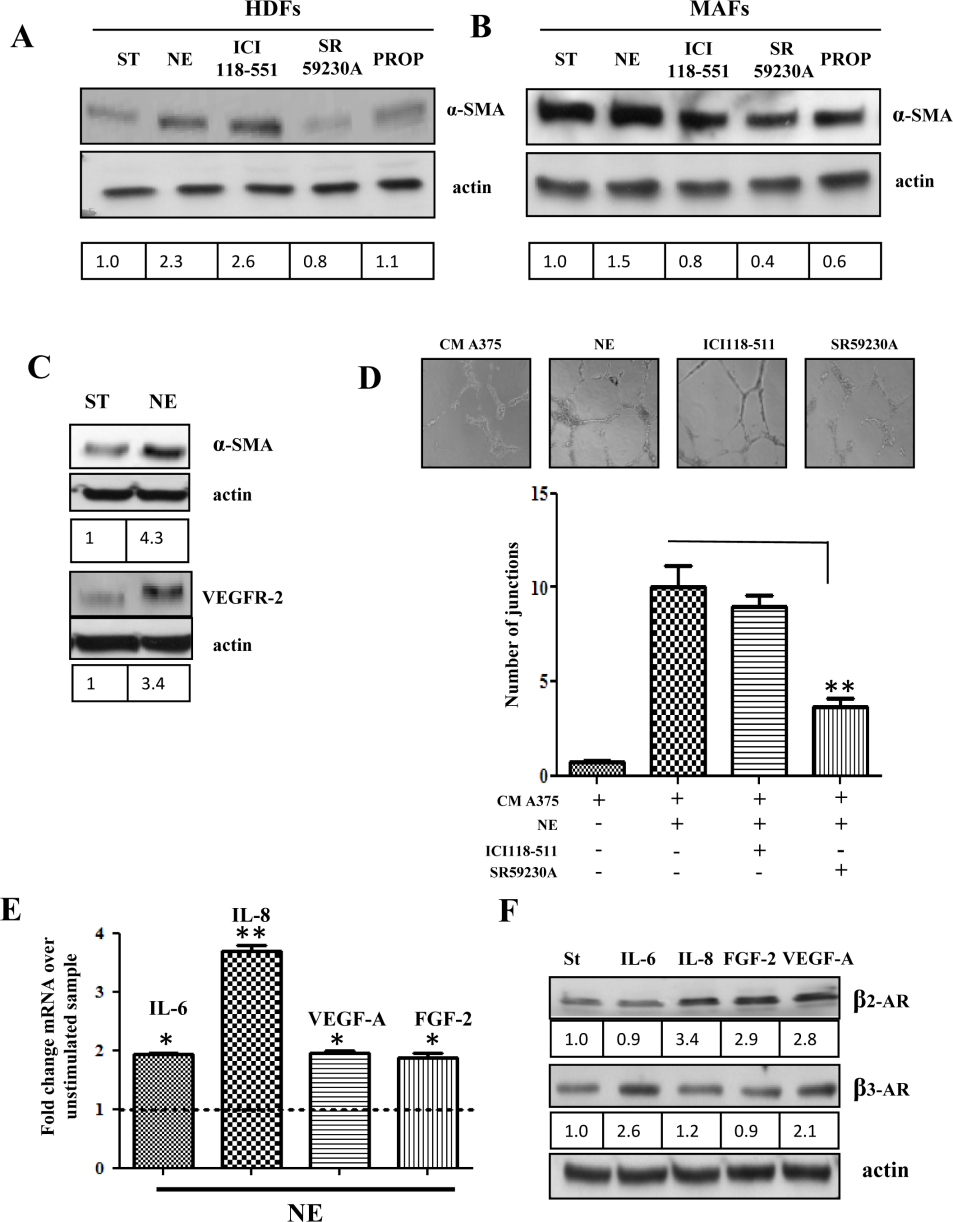
*In vitro* activation of stromal cells by NE HDFs **(A)** and MAFs **(B**) were serum starved for 24 h and then incubated in the presence or absence of NE (10 μM) alone and in combination with β-ARs antagonist ICI118-551 (1 μM), SR59230A (10 μM) and propranolol (1 μM) for additional 24 h, then αSMA levels were revealed in total lysates by immunoblotting. Anti-actin antibody was used to ensure equal protein loading. **(C)** MSCs were serum starved for 24 h and then incubated in the presence or absence of NE (10 μM) for additional 24 h. αSMA and VEGFR-2 levels were analyzed by immunoblotting. Anti-actin immunoblot was used to ensure equal protein loading. **(D)** MSCs were serum starved for 24 h, seeded in Matrigel-coated dishes and incubated with CM from HDFs, treated for 24 h in the presence or absence of NE (10 μM) and/or βARs antagonists ICI118-551 (1 μM), SR59230A (10 μM), **P < 0.001 *vs* NE stimulated cells. After 24 h formed capillary were photographed and the number of junctions were counted in 6 fields. Pictures are representative of three independent experiments. **(E)** HDFs were serum starved for 24 h and then incubated with NE (10 μM) for additional 24 h. *IL-6*, *IL-8*, *VEGF-A* and *FGF2* transcripts were evaluated by quantitative Real-Time PCR as described in Material and Methods. Data represent the mean of three independent experiments and are shown as fold change compared to untreated HDFs, *P < 0.05, **P < 0.001. **(F)** A375 cells were serum starved for 24 h and then incubated in the presence or absence of IL-6 (50 ng/ml), IL-8 (50 ng/ml), VEGF-A (50 ng/ml) and FGF-2 (50 ng/ml) for additional 24 h. β3 and β2 AR expression was evaluated in total lysates by immunoblotting. Anti-actin immunoblot was used to ensure equal protein loading. Figure is representative of three independent experiments.

CAFs have been shown to undergo an activation due to contact with cancer cells, consisting in their ability to behave as myofibroblasts, contracting collagen fibers through expression of α-smooth muscle actin (α-SMA) and secreting several pro-inflammatory and proangiogenic cytokines, such as IL-6, IL-8, VEGF-A, FGF-2 [24, 26]. Both HDFs and MAFs were analyzed for their activation in response to NE treatment. The results indicate that: *i)* MAFs show a strong basal α-SMA expression, suggesting an activation of these fibroblasts due to the fact that they are derived from aggressive cancers, *ii)* both HDFs and MAFs react to NE by increasing their expression of α-SMA, although MAFs are less sensitive (Fig. 4A, B). Importantly, β3-ARs appear to play the main role in the regulation of stromal reactivity, as revealed by β2/β3/α1-AR selective antagonists on α-SMA expression (Fig. 4A, B, Supplementary. Fig. 4C).

MSCs recruited to tumor sites have been shown to escort resident CAFs, as well as endothelial cells in their protumoral function, due to their ability to differentiate into fibroblasts or to orchestrate vessel-like structures [31, 32]. Our results indicate that human bone marrow-derived MSCs respond to NE treatment with an increase in expression of both α-SMA and VEGF receptor-2 (VEGFR-2) (Fig. 4C), as well as with an enhancement of their ability to form vessel-like structures in a capillary morphogenesis assay (Fig. 4D).

Stromal reactivity due to tumor progression enhances the production of several cytokines by resident fibroblasts [33]. In keeping with this, NE is able to induces enhanced expression of IL-6, IL-8, VEGF-A and FGF-2 mRNAs (Fig. 4E), thereby indicating that the acknowledged ability of fibroblasts to orchestrate *de novo* angiogenesis *in vitro* and to sustain inflammation is enhanced upon catecholamine release. We also observed that IL-6 and VEGF-A are able to enhance the expression of β3-AR in melanoma cells, while β2-AR is upregulated by IL-8, FGF-2 and VEGF-A, thereby suggesting a feed-forward loop between NE and its cognate receptors β3/β2-ARs (Fig. 4F).

### β-ARs expression/activation in cancer cells enhances their invasiveness and stem-cell traits, as well as the ability to favor capillary morphogenesis of endothelial cells

**Figure 5:**
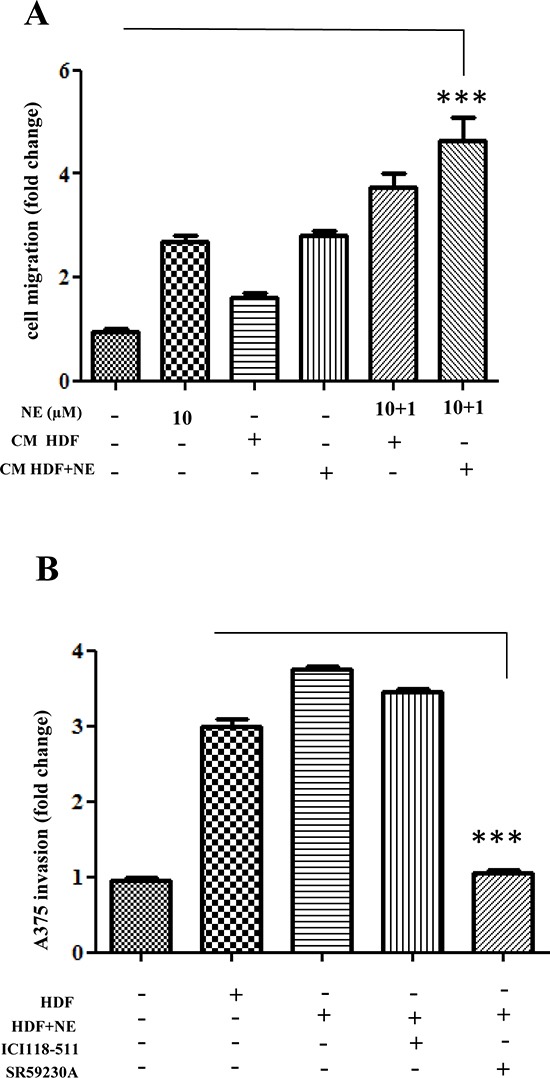
Enhancement of tumor cells invasiveness after stromal fibroblast interaction **(A)** A375 cells were serum starved for 24 h, seeded in Matrigel-coated upper side of the Boyden chambers and allowed to migrate toward CM derived from HDFs, preincubated for 24 h with NE (10 μM). The last two samples were exposed again to NE (1 μM) for additional 24 h, ***P < 0.0001 *vs* standard medium. **(B)** A375 cells were stained with Quantum Dot to render them fluorescent. After 24 h cells were serum starved for additional 24 h and seeded in the upper side of Matrigel-coated and then allowed to invade CM derived from HDFs, preincubated in the presence or absence of NE (10 μM) and/or βARs antagonist ICI 118-551 (1 μM), SR59230A (10 μM) for 24 h. Bar graphs show the mean ± SD of three independent ***P < 0.0001 *vs* CM from HDFs-NE stimulated.

To determine the involvement of β-ARs activation within the tumor:stroma milieu, we decided to analyze *in vitro* three key functions played by stromal cells, i.e. the pro-invasive and pro-angiogenic spur given by activated-stromal cells in *in vitro* co-cultures, as well as the ability to enhance stem-like traits in cancer cells. We therefore tested the sensitivity of melanoma cells to NE, to CM of HDFs activated with NE or to the combination of the two treatments. Indeed, we speculated that the combination of NE-stimulation of HDFs and of melanoma cells, once they have contacted NE-activated stromal HDFs and they have undergone enhanced expression of β-ARs, should maximize malignancy of melanoma cells. In this experimental setting, we assayed the ability of melanoma cells to move in a transwell assay. The results clearly indicate that HDFs stimulated with NE elicit a motile spur in melanoma cells (Fig. 5A), likely due to the ability of NE to activate fibroblasts to myofibroblast-like cells. Furthermore, the pro-migratory effect of NE on melanoma and on HDFs is additive (Fig. 5A), suggesting a wide function of NE stimulation in the melanoma:stroma context.

To correlate this NE-driven mobility to melanoma aggressiveness, we also carried out invasion assays of melanoma:fibroblasts co-cultures. Quantum-dots-labelled melanoma cells were kept in co-culture for 48 h with HDFs and stimulate with NE. The results indicate that the contact with HDFs enhances the invasive ability of melanoma cells (Fig. 5B). The involvement of specific β-ARs has been addressed using the specific β-blockers. Noteworthy, SR59230A is able to completely revert the ability of CAF contact to elicit invasiveness, while ICI118-551 is mostly ineffective (Fig. 5B).

**Figure 6:**
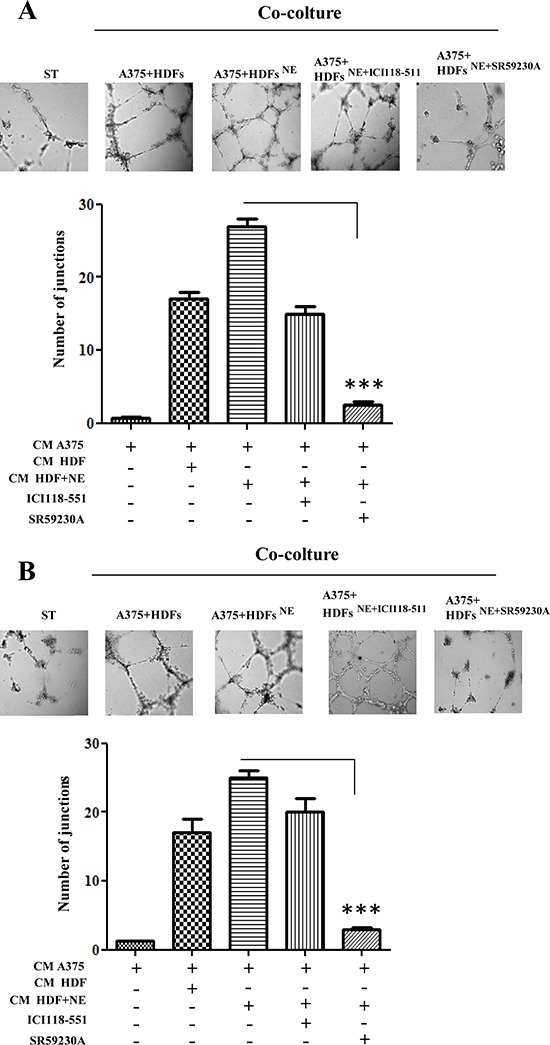
Endothelial cells reactivity HUVECs **(A)** and EPCs **(B)** were serum starved for 24 h, seeded in Matrigel-coated dish and incubated with CM derived from A375 cells, co-cultured with HDFs, preincubated in the presence or absence of NE (10 μM) and/or β-ARs antagonist ICI 118-551 (1 μM), SR59230A (10 μM) for 24 h. After 24 h, pictures of cells that formed capillaries were taken and number of junctions were counted in 6 fields. Pictures are representative of three independent experiments, ***P < 0.0001 *vs* CM from A375 and HDFs-NE stimulated.

Second, we also assayed the capillary morphogenesis of HUVECs and EPCs upon contact with NE-treated melanoma:fibroblasts co-coltures. Conditioned medium from NE-treated co-cultures of melanoma cells:HDFs were used in a capillary morphogenesis assay, as an indication of endothelial cells commitment towards tube-like structures-forming cells. We used mature endothelial cells as HUVECs and EPCs, isolated from the blood of 3 human umbilical cords and immunophenotyped by flow cytometry [34]. Mature endothelial cells have been reported to drive *de novo* angiogenesis, while EPCs have been correlated with vasculogenesis, organized by tumor-recruited precursors [35]. The results show that NE strongly enhances the pro-angiogenic properties of melanoma:HDFs co-cultures (Fig. 6A, B). The involvement of β-ARs has been addressed using the specific β-blockers ICI-118-551 and SR59230A. Both antagonists decrease tube-like structure formation for both mature and precursors endothelial, although with a main implication of β3-ARs (Fig. 6A, B).

**Figure 7:**
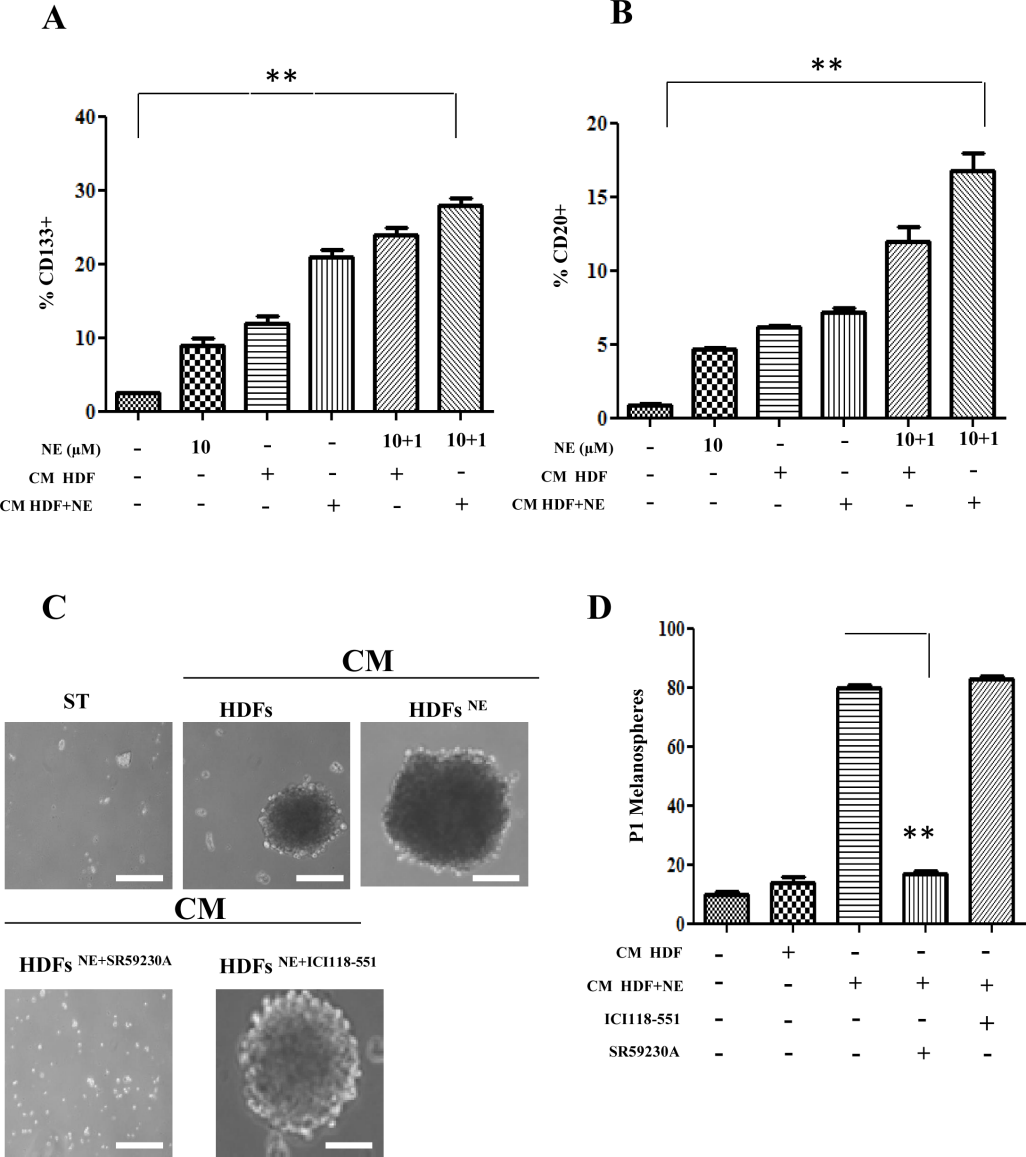
Cancer stemness induced by NE and HDFs **(A, B)** Serum-starved A375 cells were stimulated for 24 h with CM derived from HDFs, incubated in the presence or absence of NE (10 μM) and/or β-ARs antagonist ICI 118-551 (1 μM), SR 59230A (10 μM). The last two samples were exposed again to NE (1 μM) for additional 24 h. Stem cells marker CD133 **(A)** and CD20 **(B)** were analysed by cytofluorimetric analysis **P < 0.001 *vs* NE stimulated cells. **(C, D)** A375 cells were serum starved for 24 h and incubated with CM derived from HDFs, preincubated with βARs antagonist ICI 118 (1 μM), SR59230A (10 μM). Cells were incubated in low-attachment dishes to evaluate melanospheres formation. Scale bars represent 100 μm. **(C)** After 10 days, pictures of P0 melanospheres were taken and representative images are shown. **(D)**, P1 individual spheres derived after 10–15 days cultured cells, dissociated from single P0 melanospheres, were counted. Bar graphs show the mean ± SD of three independent experiments **P < 0.001 *vs* CM from HDFs-NE stimulated.

Besides motile and vessel forming ability of melanoma cells in response to NE administration, we also investigated whether NE administration could induce stem-like traits in cancer cells. The results indicate that NE is able to increase expression of stemness markers in melanoma cells, such as CD20 and CD133 (Fig. 7A, B). P_0_ and P_1_ melanosphere formation is an acknowledged feature of anchorage-independent stem cells. NE administration is able to enhances both melanosphere forming ability of A375, therefore supporting the role of NE as a stem-like promoting factor in melanoma (Fig. 7C, D). The use of selective β-blockers suggest that β3-AR is the main receptor involved in the melanosphere self-renewal ability of A375 cells.

These findings collectively suggest that β3-AR affects melanoma malignancy by acting on both cancer cells and stromal populations *in vitro*, coordinating angiogenic responses, as well as motility and stem-cell traits.

## DISCUSSION

Here we report that β-ARs are key molecular players of melanoma aggressiveness and that their function is not restricted to cancer cells, but these receptors are strongly expressed and actively functional in a large set of tumor associated cells, such as cancer associated fibroblasts, macrophages and endothelial cells. Indeed, both β-ARs are overexpressed in melanoma, with a clear correlation with malignancy. β3-ARs appear to be the main responsible for instructing melanoma cells to respond to environmental cell signals, to sense cancer associated fibroblasts and macrophages enhancing their motility and stem-like traits. Moreover, several accessory cells also express β3-AR and its functional activation plays an important role in eliciting stromal reactivity, to sustain secretion of proinflammatory cytokines and to drive *de novo* angio/vasculogenesis. All these events are promoting melanoma aggressiveness. Accordingly, patients-derived tissue showed that β3-ARs is expressed in stromal, inflammatory and vascular cells and this expression positively correlates with melanoma malignancy.

It is widely accepted that tumor cells do not proliferate and progress as isolated entities. In fact, they rely on microenvironment support, which include extracellular matrix and several supporting stromal cells. Cancer cell microenvironment is a strong determinant for metastatic potential [23, 24, 27]. Thus, during primary tumor formation, tumor cells engage a complex collection of mesenchymal cells, jointly forming the tumor-associated stroma [31]. As tumor progression proceeds, the stromal cells create a “reactive stroma” that releases a variety of signals that induce changes in cancer cells phenotype. This reactive stroma consists in CAFs, converted into myofibroblasts by cancer cell-released factors, endothelial cells forming blood-and lymph vessels, as well as inflammatory cells (i.e. cancer associated macrophages) that are recruited to tumor by cancer secreted chemokines. All these host cells engage a continuous molecular cross-talk with cancer cells, secreting large amounts of factors and cytokines that ultimately will influence invasion ability and metastasis. Stromal cells can be resident, i.e. activated mesenchymal/endothelial cells already present within the tissue where the tumor occur, or recruited by circulating precursors, mainly derived from bone marrow-derived stem cells or monocytes [36–38]. Once activated by tumor-released factors, or recruited by circulating precursors, these stromal cells are able to exert protumoral effects. Indeed, in several cancer models CAFs have been reported to induce invasiveness, resistance to apoptosis and chemotherapic agents, as well as to enhance the stem-cell traits of cancer cells, thereby contributing to metastatic spread [39]. Herein, we include NE within the large set of soluble factors that can enhance stromal reactivity and recruit bone marrow precursors (MSCs) to tumor site. So far these characteristics were restricted to the pro-inflammatory cytokines IL-6, TGF-β and SDF-1. Our findings are therefore novel and illustrate a novel interactive circuitry among melanoma progression, inflammation and stress. β3-ARs are the main effectors of MSCs recruitment, their differentiation towards fibroblasts, as well as CAFs reactivity induction. In keeping with the role played by CAFs in other cancer models, we also observed that β3-ARs expression in CAFs is mandatory to enhance invasiveness of melanoma cells, as well as to enhance their stem-like traits (e.g. CD133 and CD20 expression and P1 melanospheres formation).

β-ARs are also involved in monocytes recruitment induced by melanoma cells with a main involvement of β3-ARs over β2-ARs. Once recruited to the tumor site, monocytes differentiate into active macrophages, where they may play a multifaceted role for tumor progression. Indeed, M1 polarized macrophages are likely to play an antitumoral response whereas M2-polarized macrophages have been associated with a chronic inflammatory response that characterizes several highly malignant *neoplasiae* [40, 41]. In particular, tumor infiltration characterized by M2-polarized macrophages has been associated with enhanced stromal fibroblasts reactivity and angiogenesis, and hence with worst prognosis in several cancers [37]. Here we reported that M2-polarized macrophages contact induces β3-ARs expression in cancer cells, whereas M1-polarized macrophages do not. It is also plausible that β3-ARs could play a key role in the management of the overall inflammatory response within melanoma site. Indeed, catecholamine stimulation could enhance melanoma cells release of cytokines, such as IL-6, IL-8, VEGF-A and FGF-2, that sustain inflammatory, angiogenic and motility tasks. In turn, these cytokines are active on melanoma cells to enhance expression of β2-AR and β3-AR, thereby closing the circuitry.

Our findings clearly indicate that β-ARs are also involved in endothelial cells functions. First, the contact with mature endothelial cells or bone marrow derived endothelial precursors is able to induce β3-ARs expression in melanoma cells, while β2-AR levels appear to be unchanged. Catecholamine stimulation is able to activate the recruitment by melanoma cells of both mature and precursors endothelial cells with a clear implication of β3-AR. *In vitro* capillary morphogenesis assays suggest that β3-ARs are likely mediators in both angiogenesis and vasculogenesis function, although the contribution of β2-ARs is also relevant. In keeping with the reported role of stromal fibroblasts in orchestrating *de novo* angiogenesis in several cancer models [42–44], HDFs activated by catecholamine administration drive this β3-AR-dependent angiogenic response. Hence, there is a clear synergy between tumor accessory cells (e.g. fibroblasts and macrophages) and endothelial cells, possibly sustained by the cross-talk among stress and inflammatory factors, driving stromal reactivity and new vessels formation. Both these last features are likely exploited by malignant cells to succeed metastases.

Stress delivered catecholamines can reach the tumor mass *via* circulating blood or *via* local sympathetic nerve fibers [12, 45, 46]. In support of our data it has been shown that chronic stress enhances progression of acute lymphoblastic leukemia via β-adrenergic signaling and β-blockers can inhibit tumor burden and dissemination [12]. Importantly, the authors speculated that β-adrenergic signaling may affect the interplay between cancer cells and immune or microenvironmental cells, but they did not provide mechanistic insight. Additionally, in a model of pancreatic cancer, activation of β-adrenergic signaling mimics effects similar to chronic stress, and pharmacological β-block reverts these effects [47]. Our results of β-ARs activation in tumor accessory cells are novel and open new perspective for pharmacological intervention to prevent melanoma metastases promoted by stress. Indeed, several preclinical and observational studies have suggested the possibility that β-ARs blockers, drugs originally intended for the treatment of cardiovascular disease, may provide new therapeutic opportunities for the control of cancer progression, including that of melanoma, breast and prostate cancers [48]. Notably, a great reduction in the risk of disease progression with β-blockers has been reported for melanoma patients [18, 49, 50]. Although these studies have been recently questioned [51, 52], they promisingly suggest a potential role for targeting the β-AR pathway in melanoma patients, indicating the need for randomized clinical trials. We herein emphasize two key aspects that should be taken into account for pharmacological intervention on melanoma: *i)* β3-AR plays an important role in melanoma cell aggressiveness suggesting selective β3-AR antagonists as important agents in reducing stromal fibroblasts reactivity, *ii)* non neoplastic stromal cells may be also targeted by this therapeutic regimen, with the interesting consequence to avoid the troublesome resistance to therapy, which is typical of cancer cells.

## MATERIALS AND METHODS

### Materials

Unless specified, all reagents were obtained from Sigma; all cytokines used were from Peprotech.

### Immunohistochemistry

Forty melanocytic lesions from 40 patients were evaluated. Tissue samples were retrieved from the archives of the Divisions of Pathology of Pistoia Hospital, Pistoia, and of Pathological Anatomy, Department of Surgery and Translational Medicine, University of Florence. One sample from each case was analysed. The protocol has been approved by the Institutional Review Board for the use of human tissues. The study series included 5 common melanocytic nevi (CN); 5 atypical melanocytic nevi (AN); 5 *in situ* primary melanoma (ISM); 9 superficial spreading melanoma (SSM) (thickness 0.30-1.90 mm, mean 0.82 mm; 5 level II, 3 level III); 6 nodular melanoma (NM) (thickness 1.40-17 mm, mean 5.2 mm; 2 level III, 3 level IV, 1 level V), 10 metastatic melanoma (MM), 5 cutaneous and 5 lymph-nodal.

Sections of 4 μm thickness were cut from tissue blocks of formalin-fixed, paraffin-embedded samples. Immuno-staining was performed according to standard procedures. Briefly, antigen retrieval was performed by immersing the slides in a thermostat bath containing Epitope Retrieval Solution EDTA (pH 8.0) (Bio-Optica, Italy) for 20 min at 97°C followed by cooling for 20 min at room temperature. Endogenous peroxidase activity was blocked with 3% hydrogen peroxide in distilled water for 10 min. After blocking with normal horse serum (UltraVision, Bio-Optica, Milan, Italy), sections were incubated overnight at 4°C with the β3-AR antibody (1:100, Thermo Scientific, Milan, Italy). Signal was detected by using UltraVision Quanto Detection System AP and the bound antibody was visualized using a permanent Fast Red as chromogen (Thermo Scientific, Milan, Italy). Nuclei were counterstained with Mayer's haematoxylin. Negative control was performed by substituting the primary antibody with a non-immune serum at the same concentration. Granular layer of the epidermis, normal eccrine sweat glands and, at lesser extent, sebaceous glands, expressed β3-AR. Eccrine sweat gland staining was used as a positive internal control (47).

Immunostaining was independently assessed by two observers (D.M., S.M.). Discrepancies in the reading were resolved by a second parallel reading of the slides. The percentage of positive cells per lesion was scored according to semi-quantitative criteria. Since the percentage of positive nevus melanocytes/melanoma cells was always higher than 50%, semi-quantitative results were expressed as score 1 (50–80% positive nevus melanocytes/melanoma cells), score 2 (81–90% nevus melanocytes/melanoma cell staining), and score 3 (91–100% melanoma cell staining). The cell staining intensity was scored on a scale as weak, moderate, strong, very strong.

For statistical analysis, non-parametric tests were used to determine significant differences between groups. The percentage of positive cells per lesion after immunological staining in each group was the unit of analysis. Groups were: nevi, primary melanoma, MM. Statistical evaluation was performed comparing nevi versus malignant lesions, ISM + SSM versus NM+MM, CN versus AN. Differences were assessed using the Fisher test and were considered significant at *p* ≤ 0.05.

### Cell cultures and transfections

A375 human melanoma cells were from ATCC. Cells were grown in DMEM containing 10% FBS and 10 U/ml penicillin and 10 mg/ml streptomycin. MSCs were isolated from bone marrow aspirated of three different human volunteers, following donor consent. Cells were grown in in T75 cm^2^ with low glucose DMEM (1 mM glucose). Experiments under hypoxic conditions (1% O_2_) were performed in the hypoxic incubator. Human monocytes were obtained from normal donor buffy coat by gradient centrifugation using Ficoll and macrophages were polarized as described by Comito and colleagues [37]. For transient transfections, A375 cells were plated in 60-mm cell culture dishes and grown to 80% confluence. The siRNA (Life Technologies) was diluted to a final concentration of 20nM. Transfections were performed using Lipofectamine (Invitrogen), following manufacturer's recommendations.

### Isolation of human dermal fibroblasts (HDFs) and melanoma associated fibroblasts (MAFs)

HDFs were isolated from healthy leg regions of volunteers. MAFs were isolated from patients diagnosed with primary melanoma placed in the back dorsal zone. A small piece of the tumor tissue was minced into <1 mm pieces in size and digested overnight in 1 mg/mL collagenase I at 37°C. They were then spun down, re-suspended and plated into complete medium. We also excluded contamination by skin stem cells of HDFs, by evaluation of CD34 and cytokeratin-15, established skin stem cell markers [53].

### Endothelial cells isolation and characterization

Human umbilical cord blood from healthy newborns with a number of total nucleated cells <1.5 × 10^9^ was chosen in accomplishment with rules of Umbilical Cord Bank of Careggi Hospital (Florence, Italy), with maternal informed consent, in accordance with the Declaration of Helsinki and in compliance with Italian law. Endothelial precursors cells (EPCs) have been isolated from Umbilical Cord Bank as reported in Margheri et al [54]. Briefly, blood was diluted 1:1 with Hanks balanced salt solution and was overlaid on an appropriate volume of density gradient separation medium. Cells were centrifuged for 30 minutes at room temperature at 740*g*. Mononuclear cells were recovered, washed 3 times with Hanks balanced salt solution and resuspended in complete EGM-2 medium, supplemented with 10% FBS, 50 ng/mL VEGF, and 5 IU/mL heparin. Cells were seeded on gelatin-coated 6-well tissue culture plates at a density of 5 × 10^5^ cells/cm^2^ in a 5% CO_2_ humidified incubator at 37°C. On days 4 and 7, half of the medium was exchanged with fresh medium. Then the medium was changed completely with EGM2–10% FBS every 3 days. EPC colonies appeared in cell cultures after 2–3 weeks and were identified as circumvented monolayers of cobblestone-like cells. EPCs were analyzed for the expression of surface antigens by flow cytometry (CD45, CD34, CD31, CD105/R, VEGF-R2).

### Preparation of conditioned media

Conditioned media (CM) were obtained from HDFs, MAFs, MSCs or macrophages. Cells were grown to sub-confluence, then serum starved and incubated for 48 h before collection of the CM. CM were harvested, clarified by centrifugation, and used freshly.

### Western blot analysis

Cells derived from our experimental conditions were lysed for 20 min on ice in 500 μl of RIPA lysis buffer. 20 μg of total proteins were loaded on SDS–PAGE, separated and transferred onto nitrocellulose (Millipore). The immunoblots were incubated in 3% bovine serum albumin, 10 mmol/L Tris–HCl (pH 7.5), 1 mmol/L EDTA, and 0.1% Tween 20 for 1 h at room temperature, probed first with specific antibodies and then with appropriate secondary antibodies.

### *In vitro* boyden invasion assay

Transwells (Costar), equipped with 8-μm-pore polyvinylpyrrolidone-free polycarbonate filters, were coated with 50 μg/cm^2^ of reconstituted Matrigel (BD). Cells were loaded into the upper compartment (5 × 10^4^ cells in 200 μl) in serum-free growth medium. Cells were allowed to migrate towards complete growth medium or medium conditioned by stromal cells. After 24 h of incubation at 37°C, non-invading cells were removed mechanically using cotton swabs, and the membrane was stained with Diff-Quick solution and chemoattracted cells were counted.

### Capillary morphogenesis assay

All the experiments were performed using growth factor–reduced Matrigel at a concentration of 1 mg/mL. Fifty microliters of Matrigel was added to each well of a 96-well plate and then placed in a humidified incubator at 37°C for 30 min. HUVECs and EPCs (2 × 10^4^ cells/well) were added to the Matrigel-coated plates in a final volume of 200 μL. The effects on the morphogenesis of endothelial cells were recorded after 6 h with an inverted microscope (Leitz DM-IRB) equipped with CCD optics and a digital analysis system. Results were quantified by measuring the joint numbers in the field.

### Real time PCR

Total RNA from cells was isolated using RNeasy plus Mini Kit (Qiagen). 1 μg of total RNA was used for Reverse transcription using QuantiTect reverse transcription Kit (Qiagen). Measurement of gene expression was performed by quantitative real-time PCR (7500 Fast Real-Time PCR System, Applied Biosystems) using Quantifast SYBR Green PCR (Qiagen). The amount of target is normalized to an endogenous reference (GAPDH). The primers for VEGF-A were: 5′TTCTGCTGTCTTGGGTGCAT-3′ (forward), 5′TGTCCACCAGGGTCTCGATT-3′ (backward). The primers for IL-8 were 5′-CTGGCCGTGGCTCTCTTG-3′ (forward), 5′-TTAGCACTCCTTGGCAAAACTG-3′ (backward). The primers for IL-6 were 5′AGTTCCTGCAGTCCAGCCTGAG-3′ (forward), 5′TCAAACTGCATAGCCACTTTCC-3′ (backward) bFGF, 5′-GGCGTGTACATGTGGTCTCAGA-3′ (forward) and 5′-TTATGGCTCAC TGCAACCTTGA-3′ (backward).

### Melanosphere formation

A375 cells incubated for 72 h with CM were detached using Accutase (Sigma). For melanospheres formation, single cells were plated at 150 cells/cm2 on low attachment 100 mm plate (Corning) in DMEM/F12 (Invitrogen, Carlsbad, CA, USA) supplemented with N2 (Invitrogen), 20 μg/ml insulin, 10 ng/ml bFGF and 10 ng/ml EGF. Cells were grown under these conditions for 15–20 days and formed non-adherent P0 spheres. For the evaluation of self-renewal a single melanosphere was dissociated in single cells with Accutase and a dilution of one cell per well into 96-well low attachment plates was performed in order to isolate individual P1 melanospheres.

### Flow cytometry

1 × 10^6^ A375 cells were labelled with FITC-anti-CD133 (human clone: AC133) and PE-anti-CD20 [EP459Y] antibodies for 1h at 4°C in the dark. Cells were washed and flow cytometry was performed using a FACSscan (BD Biosciences).

### Statistical analysis

Data are presented as means ± SD from at least three independent experiments. Statistical analysis of the data was performed by Student's t test. *P*-values of ≤ 0.05 were considered statistically significant.

## SUPPLEMENTARY FIGURES


