# Supplementary material for: Norepinephrine promotes tumor microenvironment reactivity through β3-adrenoreceptors during melanoma progression
Source: Oncotarget. 2014 Dec 6;6(7):4615–32. doi: 10.18632/oncotarget.2652 (PMC4467103; doi:10.18632/oncotarget.2652)
Supplement: Supplementary file 1 [file oncotarget-06-4615-s001.pdf]

SUPPLEMENTARY FIGURES

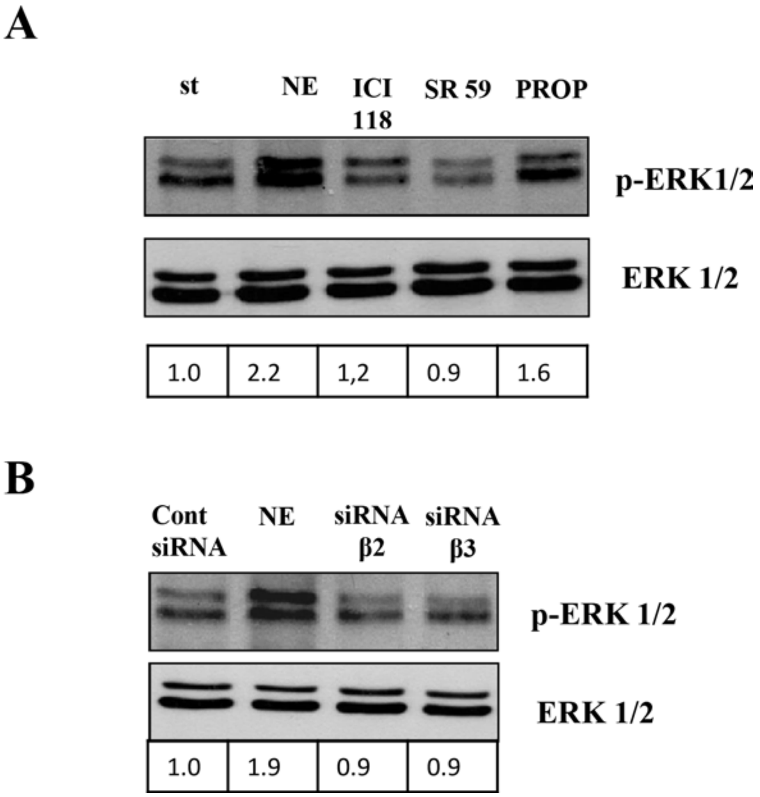

Supplementary Figure S1: A375 cells were serum starved for 24 hrs and than incubated in the presence or absence of NE (10 µM) with or without β-ARs antagonists ICI 118 (10 µM), SR59230A and propranolol (1 µM) for additional 24 hrs. Western blotting for phosphorilation of ERK and total ERK was performed. Total ERK were performed as control.

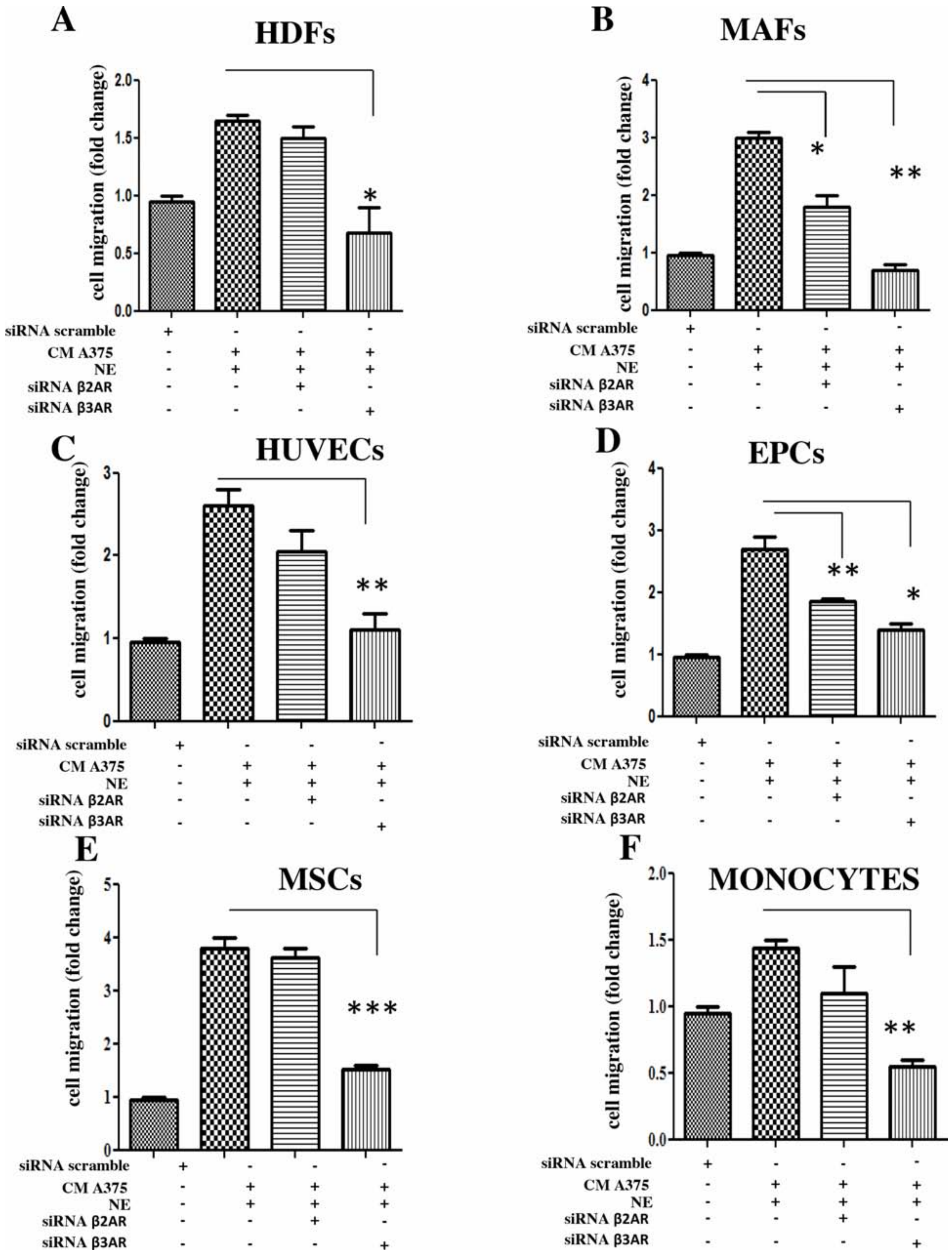

Supplementary Figure S2: Different stromal cells were allowed to migrate for 24 h toward CM derived from A375 melanoma cells subjected to siRNA silencing as indicated in the figure and described in Material and Methods. Figure shows recruitment of HDFs **(A)**; MAFs **(B)**; HUVECs **(C)**; EPCs **(D)**; MSCs **(E)** and monocytes **(F).**

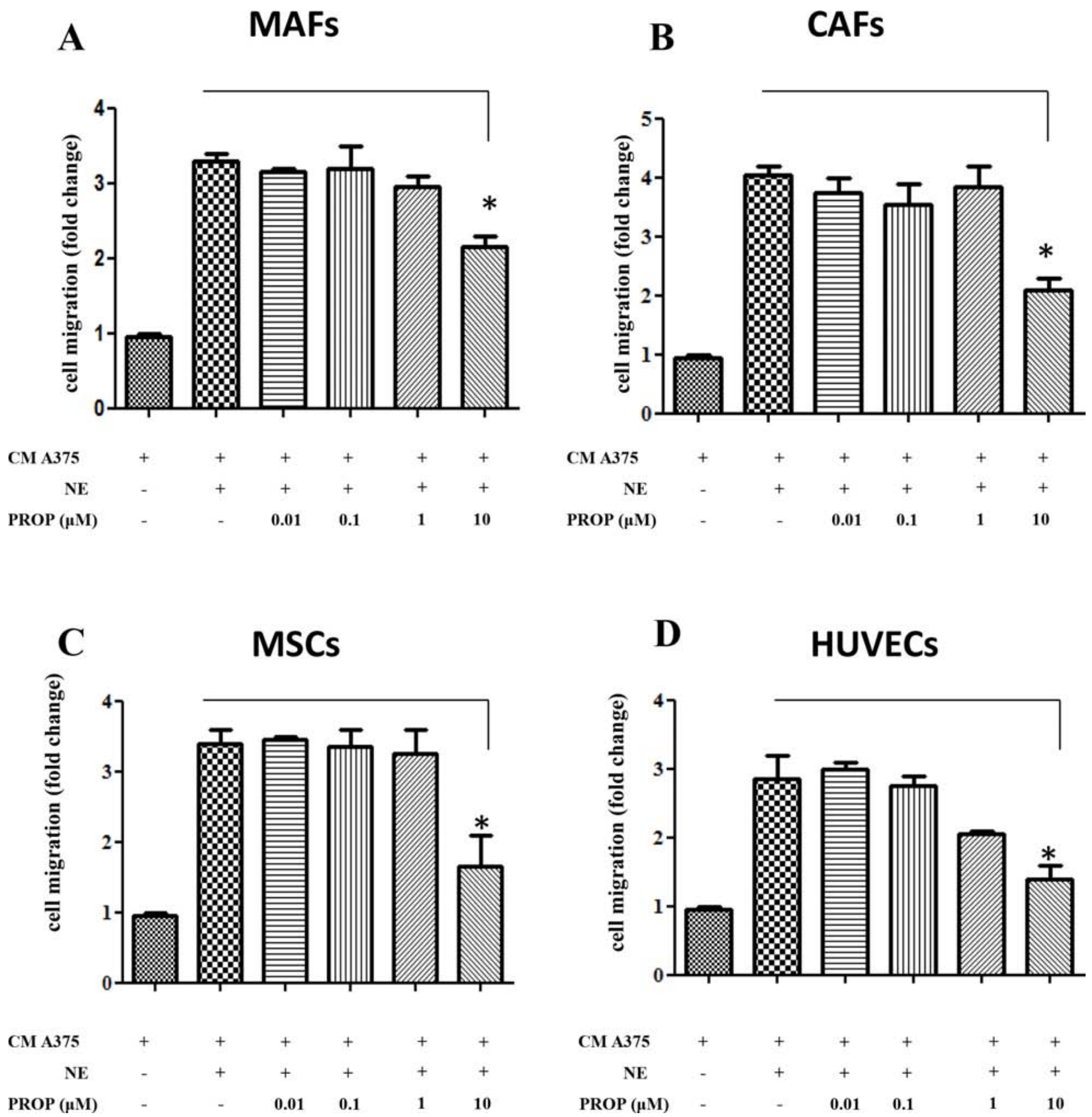

Supplementary Figure S3: Different stromal cells were allowed to migrate for 24 hrs toward CM derived from A375 melanoma. A375 cells were than incubated in the presence or absence of NE (10 µM) and/or β-ARs antagonist propranolol at different concentration ranged from 0.01 µM to 10 µM. Figure shows recruitment of MAFs (**A**)**;** HDFs **(B);** MSCs **(C);** HUVECs **(D).**

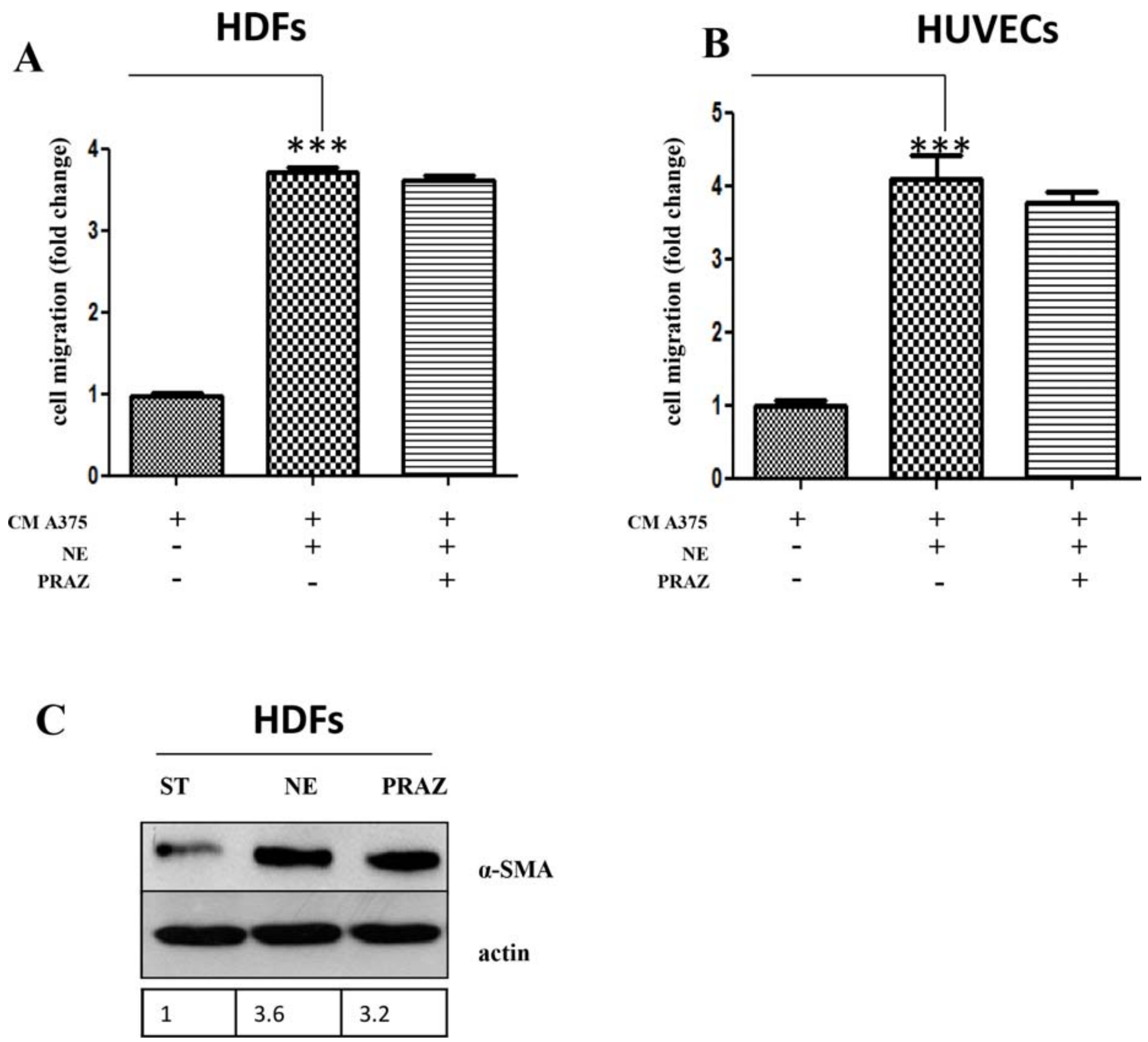

Supplementary Figure S4: HDFs and HUVECs were allowed to migrate for 24 hrs toward CM derived from A375 melanoma incubated in the presence or absence of NE (10 µM) with or without α1ARs antagonist prazosin (10 µM). Figure shows recruitment of HDFs **(A)**; HUVECs **(B)**. HDFs **(C)** were serum starved for 24 hours and then incubated for additional 24 hours in the presence or absence of NE (10µM) with or without α1ARs antagonist prazosin (10 µM). Expression of α-SMA was performed by immunoblottig. Actin was shown to ensure equal loading of protein.
